# Differential co-expression analysis reveals a novel prognostic gene module in ovarian cancer

**DOI:** 10.1038/s41598-017-05298-w

**Published:** 2017-07-10

**Authors:** Esra Gov, Kazim Yalcin Arga

**Affiliations:** Department of Bioengineering, Marmara University, 34722 Goztepe, Istanbul Turkey

## Abstract

Ovarian cancer is one of the most significant disease among gynecological disorders that women suffered from over the centuries. However, disease-specific and effective biomarkers were still not available, since studies have focused on individual genes associated with ovarian cancer, ignoring the interactions and associations among the gene products. Here, ovarian cancer differential co-expression networks were reconstructed via meta-analysis of gene expression data and co-expressed gene modules were identified in epithelial cells from ovarian tumor and healthy ovarian surface epithelial samples to propose ovarian cancer associated genes and their interactions. We propose a novel, highly interconnected, differentially co-expressed, and co-regulated gene module in ovarian cancer consisting of 84 prognostic genes. Furthermore, the specificity of the module to ovarian cancer was shown through analyses of datasets in nine other cancers. These observations underscore the importance of transcriptome based systems biomarkers research in deciphering the elusive pathophysiology of ovarian cancer, and here, we present reciprocal interplay between candidate ovarian cancer genes and their transcriptional regulatory dynamics. The corresponding gene module might provide new insights on ovarian cancer prognosis and treatment strategies that continue to place a significant burden on global health.

## Introduction

Ovarian cancer remains the leading cause of death from gynecologic malignancy with an estimated death ratio of 5% in whole cancer types^1^. Five -year survival rate of women with ovarian cancer was reported as 35% in US, and the necessity in development of effective treatment strategies was emphasized since treatment of ovarian cancer with current therapies is harder than any other type of female reproductive tract cancers^2^.

The most common type of primary malignant ovarian tumors is the epithelial carcinoma that begins in the ovary tissues^3^. This carcinoma is the most dangerous of all types of ovarian cancers. Unfortunately, it is not diagnosed until the disease is advanced in stage^4^.

Over the last decade, enormous researches have been made to understand mechanisms of ovarian cancer pathogenesis and to identify diagnostic and prognostic targets. However, disease-specific and effective biomarkers were still not available, since studies have focused on individual genes associated with ovarian cancer, ignoring the interactions and associations among the gene products. On the other hand, integration of biological data at any level (gene, transcript, protein, metabolite, etc.) with biomolecular networks (i.e., gene co-expression^5^, protein-protein interaction^6^, transcriptional regulatory^7^, and metabolic networks^8^) provides valuable insights on elucidation of the disease mechanisms^9, 10^ and identification of molecular signatures of human diseases^11–14^.

Co-expression networks are reconstructed from gene expression data using pairwise correlation metrics^5^. Altered co-expression patterns of genes between two states (for instance, healthy vs. tumor) are called differential co-expression^15^, which represent significant potential to identify gene clusters affected by state transition. Construction of differential co-expression networks and their topological analysis provide us valuable information on the alterations in biological systems in response to environmental and biological perturbations, such as disease formation and gene mutation^16, 17^. In several studies, differential co-expression networks were studied to identify disease associated genes and gene modules in human diseases including chronic lymphocytic leukemia^18^, obesity^19^, tumor-associated macrophages^20^, breast cancer^21^, and ovarian cancer^22, 23^.

Zhou and coworkers^22^ built an integrated co-alteration network via utilization of copy number, methylation and mRNA expression data, and identified 155 ovarian cancer associated genes. In another study, through weighted correlation network analysis, 3095 differentially expressed genes were identified from genome expression profiles of ovarian cancer patients in early and advanced cancer stages, and 6 prognosis related genes were selected out as novel candidates for clinical biomarkers^23^.

In the present study, considering the importance of the choice of tissue in gene expression studies^24^, we performed meta-analysis of transcriptome datasets including samples from laser micro-dissected epithelial cells in ovarian tumor and healthy ovarian tissues, differential co-expression networks were reconstructed at two different (healthy and diseased) states, and the modules (clusters of highly connected network components) of co-expression networks were comparatively analyzed. We identified a novel prognostic gene module, which was differentially co-expressed in ovarian cancer when compared to healthy tissues. Topological and functional enrichment analyses were performed to understand the molecular mechanisms. Prognostic transcriptional regulatory elements (i.e., transcription factors and miRNAs) of the module were also investigated.

## Results

### Differential gene expression in ovarian cancer

**Figure 1:**
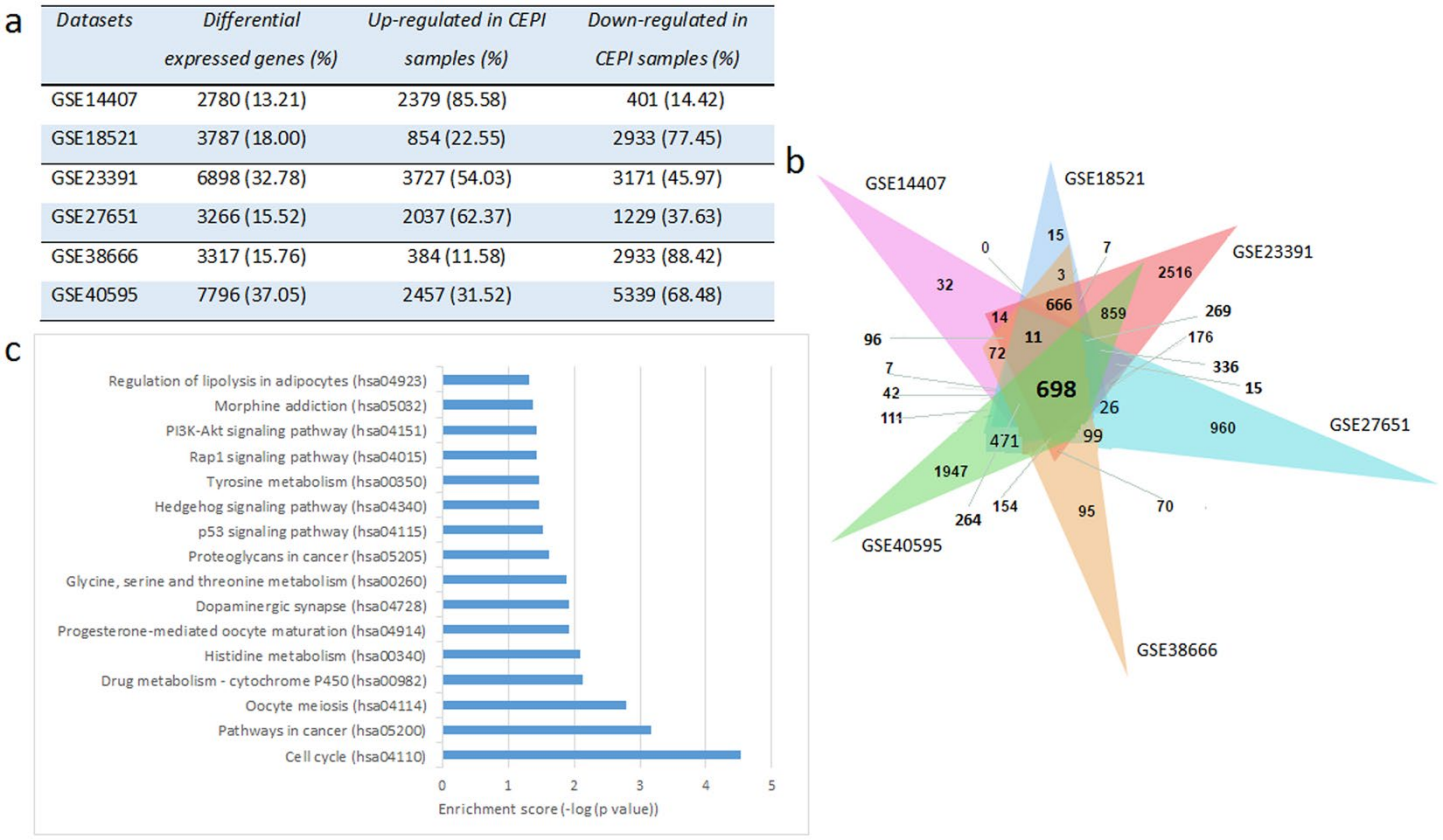
Differentially expressed genes (DEGs) in ovarian cancer datasets. (**a**) The table represents the numbers (and percentages in parenthesis) of differentially expressed genes between laser micro-dissected epithelial cells from ovarian tumor (CEPI) and ovarian surface epithelia (OSE) samples. The direction of regulation (i.e., up or down-regulation) of the genes was also specified. (**b**) Venn diagram of DEGs across the six ovarian cancer associated datasets. (**c**) Statistically significant KEGG pathways obtained through the pathway enrichment analysis of the 698 mutual DEGs of all datasets (adjusted p-value < 0.05).

In the present study, we analyzed transcriptome datasets from six independent studies associated with ovarian serous carcinoma by comparing gene expression levels between laser-micro-dissected epithelial cells from ovarian tumor (CEPI) and healthy ovarian surface epithelial (OSE) samples. To identify differentially expressed genes (DEGs) in each dataset, statistical analyses were performed, which reported statistically significant (adjusted p-value < 0.01 and fold-change >2.0 or <0.5) alterations in 13–37% of the genome (22% in average) between healthy and diseased states (Fig. 1a). Although the numbers of DEGs and their expression patterns (i.e., down or up-regulation) among datasets were incompatible, there were still 698 mutual DEGs (Fig. 1b). Functional enrichment analysis of proteins encoded by these genes indicated several biological pathways mainly associated with cell cycle, cancer signaling, drug and amino acid metabolisms (Fig. 1c).

### Co-expression profiles in ovarian cancer

**Figure 2:**
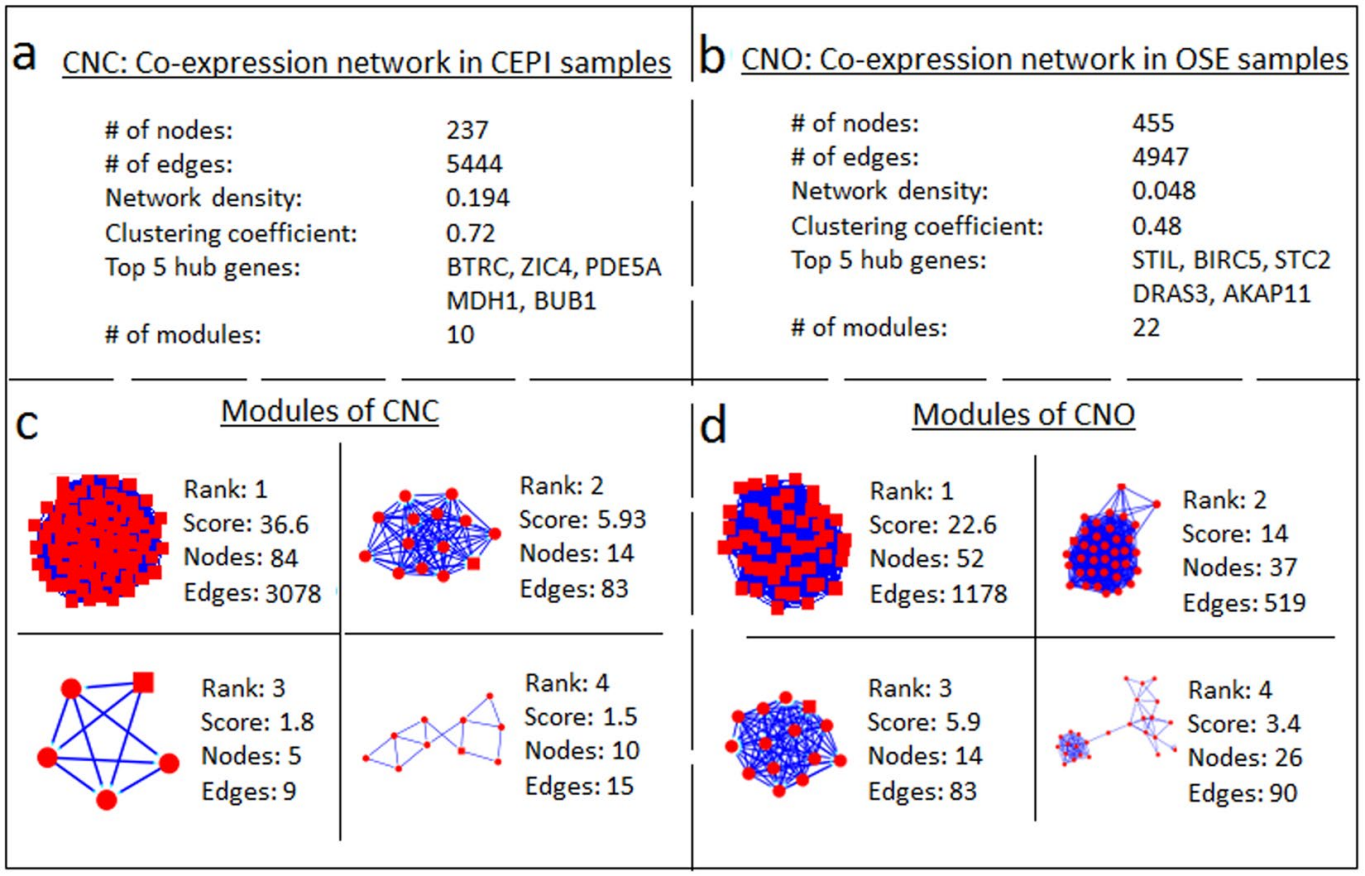
Characteristics of co-expression networks and belonging modules in laser micro-dissected epithelial cells from ovarian tumor (CEPI) and ovarian surface epithelia (OSE) samples. (**a**) Topological properties of the co-expression network in CEPI samples (CNC). (**b**) Topological properties of the co-expression network in OSE samples (CNO). (**c**) Top four co-expressed modules of CNC representing the diseased state. (**d**) Top four co-expressed modules of CNO representing the healthy state. DEGs were represented as nodes, and the statistically significant co-expression associations between DEGs were represented as edges.

Possible correlations between expression profiles of 698 mutual DEGs were identified employing Pearson correlation coefficients (PCCs). For this purpose, the expression values of mutual DEGs in diseased (CEPI) and healthy (OSE) states were employed separately. The calculated PCCs were normally distributed with a mean and standard deviation of 0.05 and 0.39, respectively (data not shown). PCC cut-offs of >0.815 (positive correlation) and <−0.815 (negative correlation), which correspond to p-value < 0.05, were employed to determine statistical significance of the pairwise correlations. Two co-expression networks (CNC and CNO) were constructed within the significantly co-expressed DEGs. CNC, which reveals the co-expression network in CEPI samples, consisted of 5444 links between 237 genes (Fig. 2a); whereas CNO, representing the co-expressions in OSE samples, consisted of 4947 links among 455 genes (Fig. 2b). When degree distributions of networks were investigated, both networks possessed scale-free topology (R^2^ > 0.80), indicating the presence of hubs. However, CNC and CNO did not share mutual hubs. Though the number of genes exhibiting co-expression pattern was almost two-fold higher in healthy state, the density (0.194) and the clustering coefficient (0.72) were higher in CNC when compared to those in CNO (0.048 and 0.48, respectively).

### Co-expressed gene modules in diseased and healthy states

To identify network modules, the topological structure of the co-expression networks (CNC and CNO) were analyzed. We identified 10 and 22 modules in CNC and CNO, respectively (Fig. 2c,d). Since we are looking for a prognostic gene module that may represent signatures in ovarian cancer pathogenesis, we focused on the modules in CNC. In addition, we expect the candidate prognostic modules to contain considerable amount of genes and represent high intensity of the connectedness within the module in order to maintain high precision in their predictive capability. Considering the topological parameters (the number of nodes, the module density and the average connectivity) of the modules in CNC (Supplementary Table S1), we selected the module with 84 genes, which also represented high average connectivity (with 36.6) and density (with 0.88), and considered in further analyses.

On the other hand, top scoring module of CNO (with 52 genes), which might give clues on the dysregulated pathways in healthy tissues, was also analyzed. However, functional enrichment analyses of this module presented expected results; the majority of the elements of the module was significantly enriched with the cell cycle pathway and cellular organization related processes (data not shown).

### The module was differentially co-expressed in ovarian cancer

**Figure 3:**
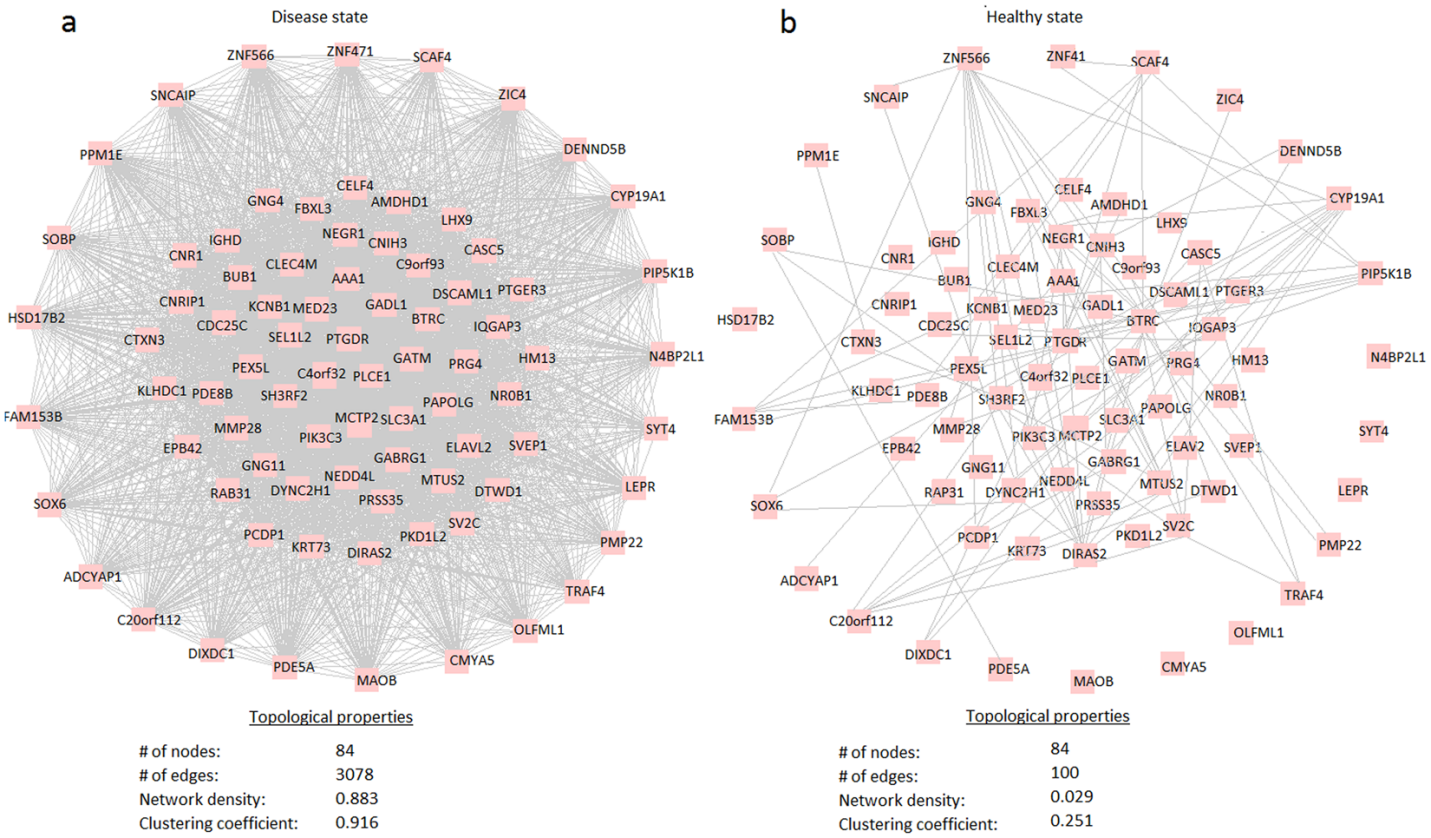
Differential co-expression module (CMC) of ovarian cancer consisting of 84 genes. (**a**) The topological features of the CMC representing the dense co-expression pattern in diseased samples. (**b**) The topological features of the CMC in healthy samples.

The selected module of CNC, so-called CMC, consisted of 3078 links among 84 genes (Fig. 3a). We also searched the CMC module within the CNO network to analyze the co-expression pattern of the module genes in the healthy state. In this case, only 100 links were observed among 48 of the 84 nodes (Fig. 3b). Furthermore, the comparative analysis of the CMC in both states indicated the significant alterations in co-expression patterns of module genes between two states. The network density in CMC was 0.883 in diseased state, whereas significantly lower (0.029) in the healthy state. These results pointed out the differential co-expression of the module in ovarian cancer. It may be suggested that the co-expression pattern among 84 genes (Supplementary Table S2) was activated in response to the change of state, and this set of genes may be considered as a “systems-biomarker” for therapeutics and prognosis in ovarian cancer.

**Figure 4:**
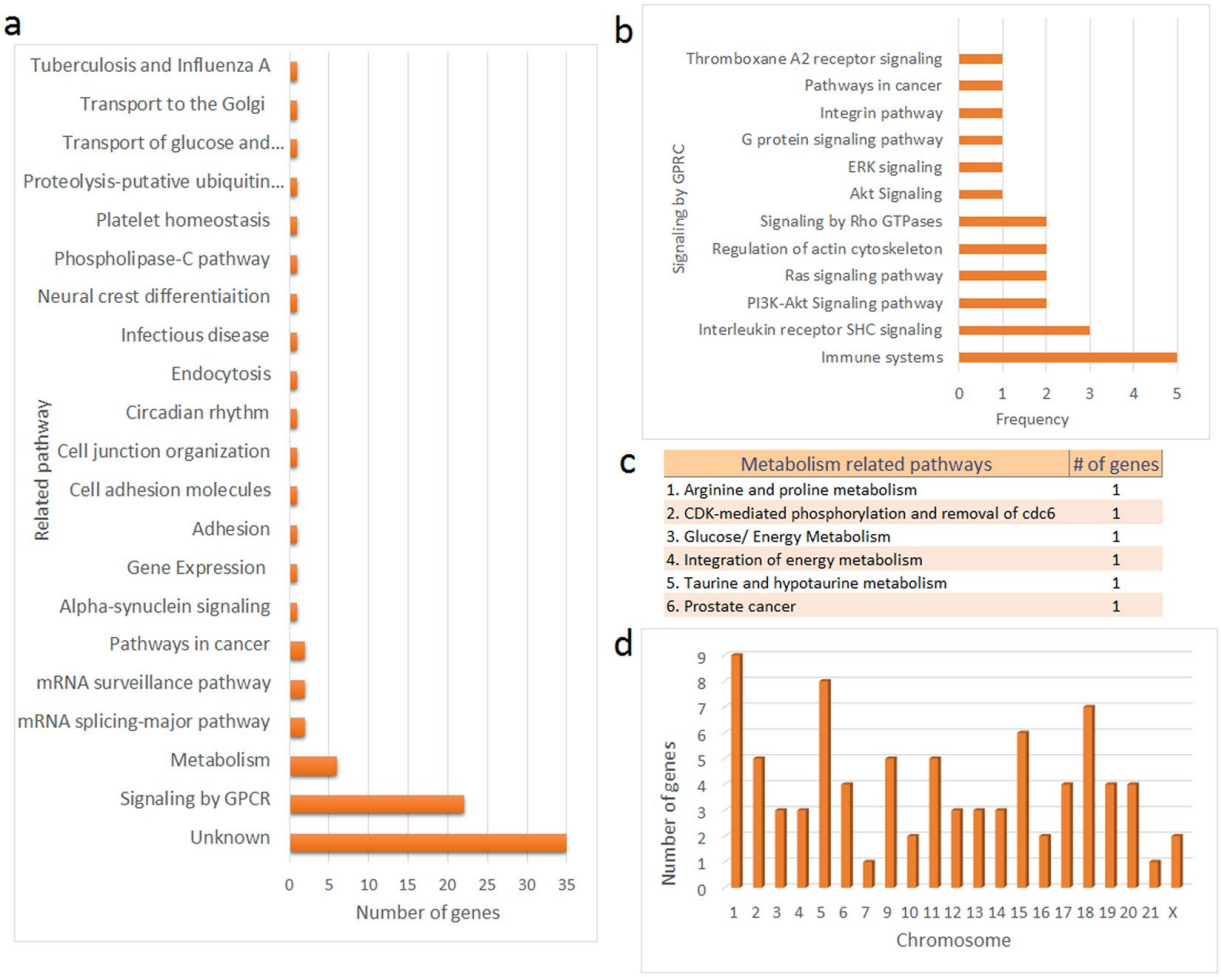
The biological processes, pathways and chromosomal locations associated with the prognostic genes. (**a**) The distribution of the prognostic genes into biological processes or molecular pathways. Process or pathway annotations of genes were obtained from GeneCards database. (**b**) The distribution of the prognostic genes into the “signaling by GPCR” subcategory. (**c**) The distribution of the prognostic genes into metabolism related pathways. (**d**) Distribution of the prognostic genes into chromosomal locations.

We performed pathway enrichment analysis on the module genes using information from several data sources including KEGG and Reactome databases. However, statistically significant enrichment results couldn’t be obtained; interestingly, the majority of these genes (90.5%) was taking roles in distinct pathways and/or not annotated in any pathway. Afterwards, to characterize the pathway or process annotation of each gene, we manually searched GeneCards Human Gene Database. A significant portion (35 genes, 42%) of the genes was not categorized in any biological pathway (Fig. 4a), whereas 22 genes (26%) were associated with GPCR signaling (Fig. 4b). When GPCR signaling pathways were investigated, 5 of 22 genes (BTRC, DYNC2H1, IGHD, NEDD4L and PIK3C3) were involved in immune systems response. In addition, 3 genes, (ADCYAP1, CDC14A, and LEPR) were taking roles in interleukin receptor SHC signaling pathway, and 6 genes (AMDHD1, CYP19A1, GADL1, GATM, HM13 and HSD17B2) were encoding proteins with metabolic activities in several biological processes (Fig. 4c). On the other hand, products of the remaining genes were distributed in several pathways or biological processes.

The module genes were not concentrated in any pathway or biological process, surprisingly. Therefore, we analyzed the chromosomal location of the genes, hypothesizing that the dense co-expression pattern of this gene set may be as a result of the co-localization of the genes on the same chromosomes. However, the genes were distributed to almost all human chromosomes (except chromosome 8 and 22) at different ratios (Fig. 4d).

### Prognostic performance of the gene module

**Figure 5:**
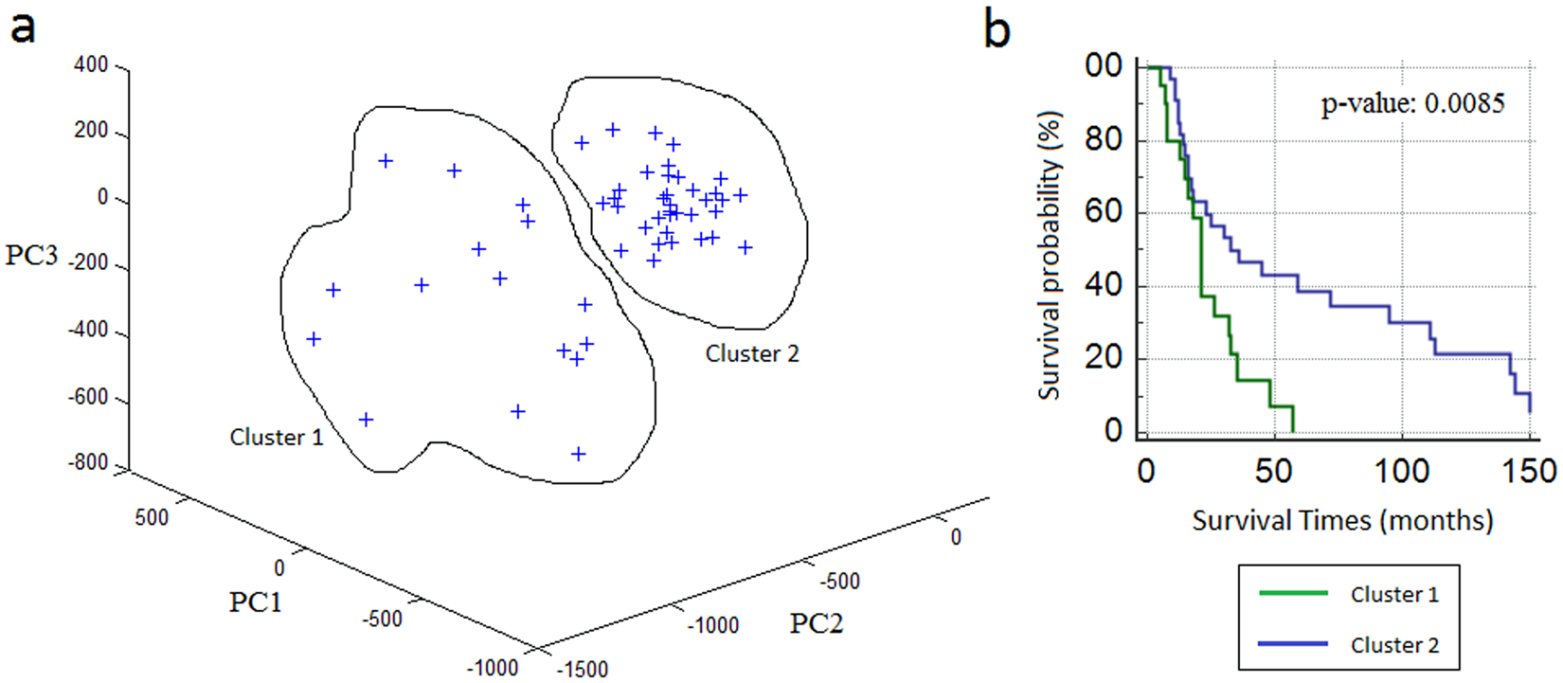
Prognostic performance of the module. (**a**) Clustering of samples using principle components (PC1, PC2, PC3) of the expression matrix of module genes. (**b**) Kaplan-Meier analysis of ovarian cancer dataset using the clusters identified through differentially co-expressed module. The p-values are computed using log-rank test.

Since the module was differentially co-expressed in ovarian cancer, and module genes were distributed in several pathways or biological processes and chromosomal locations, we hypothesized that this module may represent some common traits of ovarian cancer and can be potential marker for ovarian cancer prognosis. Therefore, we tested its prognostic capability in ovarian cancer using principle component analysis (PCA) and Kaplan-Meier survival curves. The first three principle components of the expression matrix of module genes, which describes 74.8% of the total variance, separated the samples into two clusters (Fig. 5a). Then, survival curve was plotted based on both clusters (Fig. 5b). The gene module was predictive of patient survival in ovarian cancer (p-value = 0.0085, log-rank test). Through Cox-proportional hazard analysis, and the hazard ratio was estimated as 2.36 with a 95% confidence interval of 1.21 to 4.62 (p-value = 0.0117).

### Transcriptional regulators of the module genes

**Figure 6:**
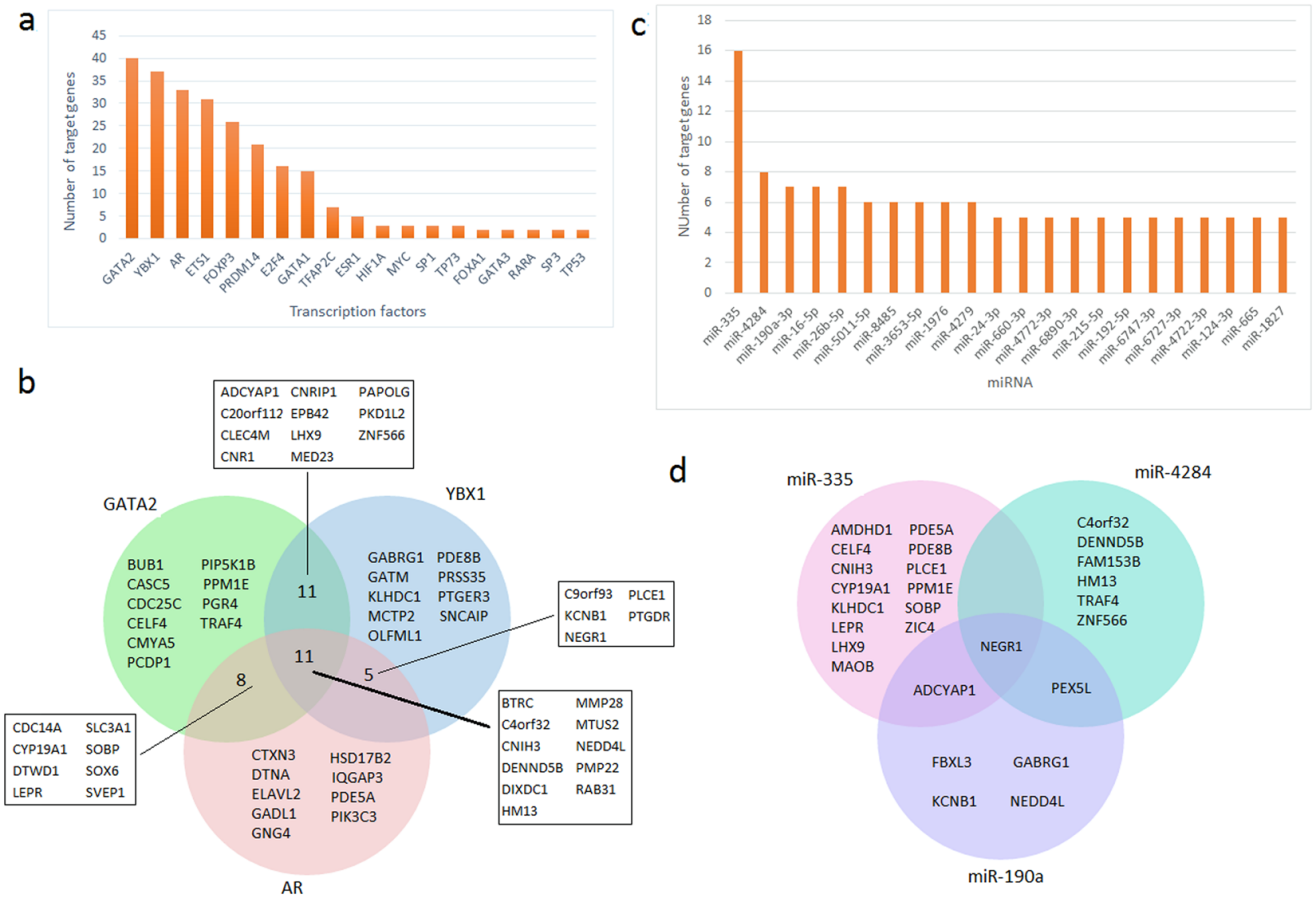
Transcriptional regulators of the prognostic genes. (**a**) Bar graph representing the distribution of transcription factors (TFs) which regulate the prognostic genes. (**b**) Venn diagram of the prognostic genes regulated by the most significant (top 3) TFs. (**c**) Bar graph representing the distribution of microRNAs (miRNAs) regulating the prognostic genes. (**d**) Venn diagram of the prognostic genes regulated by the most significant (top 3) miRNAs.

It must be revealed how accrued differential co-expression depends on conditions and which mechanisms cause these phenomena. Especially, transcriptional regulation of gene expression comes into prominence to evaluate the condition specific gene expression alterations. Transcription factors (TF) and microRNAs (miRNA) are the major regulators in transcriptional control. Therefore, relationships between TF-miRNA-module genes were investigated. It was found that six TFs (GATA2, YBX1, AR, ETS1, FOXP3, and PRDM14) were regulating the majority (89%) of module genes (Fig. 6a). In addition, 70 of 84 genes were regulated by more than one TF. Furthermore, 35 genes were co-regulated by GATA2, YBX1 and AR (Fig. 6b). When miRNA-target gene interactions were also evaluated; miR-335, miR-4284, miR-190a, miR-16, and miR-26b came into prominence with regulating at least seven target genes (Fig. 6c). Unlike TFs, miRNAs represented a diverse regulatory pattern, where module genes were regulated by distinct miRNAs (Fig. 6d).

### Differential expression of the module genes in different tumor types

**Figure 7:**
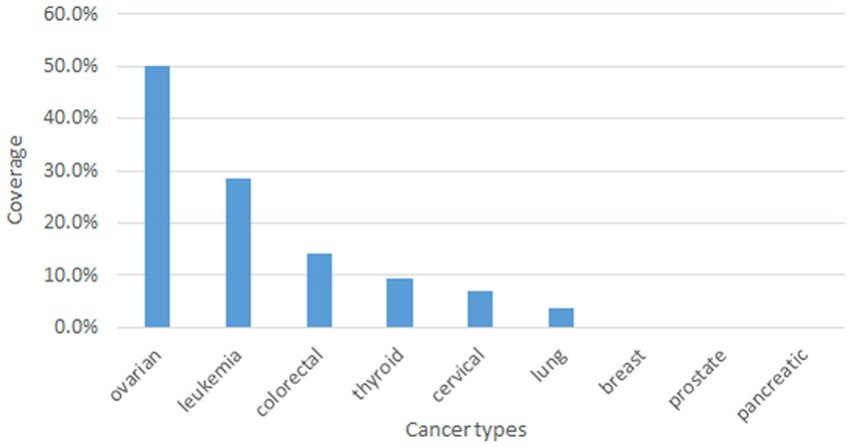
Differential expression of the prognostic genes in different tumor tissues. Bar graph represents the coverage of the module in each tumor sample, i.e. the ratio of the number of differentially expressed prognostic genes to the number of all genes in the prognostic module.

Genes of the differentially co-expressed CMC module were investigated across nine tumor tissues to determine specificity of the module to ovarian cancer, and to examine the expression pattern of the module in different tumor tissues. For this purpose, gene expression datasets associated with nine tumors (ovarian, breast, cervical, prostate, lung, colorectal, pancreatic, thyroid, and leukemia) were employed and statistically significant DEGs were identified. Module genes were screened in identified DEGs for each cancer type, and the expression pattern of the module genes in different tumor tissues were analyzed. The module genes were not detected in any of the tumor tissues as a whole (Fig. 7). However, 50% of the co-expressed module genes were determined as differentially expressed in serious ovarian tumor. Differential expressions of the genes were observed in leukemia, colorectal, thyroid, cervical and lung cancers at lower coverages (3–28%). Furthermore, none of the module genes were differentially expressed in breast, prostate and pancreatic tumor tissues. Among the module genes, BUB1, GATM, PLCE1, NEGR1, PTGER3 and SH3RF2 were differentially expressed in three or more cancer tissues (Table 1). These results indicated that the expression pattern of the module in different tumor tissues was distinct, and the module was specific to ovarian cancer.

**Table 1:** Mutual differentially expressed module genes in different tumor types.

Mutual genes	Cancer types	Regulation pattern	FC	Mutual genes	Cancer types	Regulation pattern	FC
**BUB1**	colorectal	up	12.11	**GATM**	cervical	down	3.15
	cervical	up	2.68		leukemia	up	25.34
	leukemia	down	2.43		ovarian	down	2.48
	ovarian	up	2.38		thyroid	down	3.01
**PLCE1**	colorectal	down	5.47	**PTGER3**	cervical	down	2.26
	ovarian	down	4.55		leukemia	up	3.44
	leukemia	up	2.95		ovarian	down	4.99
**NEGR1**	ovarian	down	17.44	**SH3RF2**	colorectal	down	2.54
	leukemia	up	3.18		lung	down	2.10
	thyroid	down	2.06		ovarian	down	2.89

## Discussion

Identification of alterations in co-expression patterns of genes among disease and healthy samples provides information about disease specific gene modules. Previous studies that aim to predict and prioritize candidate prognostic gene modules have focused on modules of co-expression and protein-protein interaction networks constructed around differentially expressed genes in ovarian cancer^22, 23^. However, the altered co-expression patterns of module genes during transition from healthy to diseased state were neglected in these studies. Here, we performed a differential co-expression analysis to identify disease genes and their co-expression couplings. As a result, the novel co-expressed gene module presented here might considered as “systems biomarkers” that pioneer rational design of effective strategies in ovarian cancer diagnosis and prognosis within the perspective of precision medicine^25^.

In the present study, in order to increase dimensionality and breadth of information that can be extracted, we didn’t limit the analyses with a single transcriptome dataset, but performed a meta-analysis of various transcriptome datasets. In addition, a single type of microarray was chosen as the investigation platform (i.e. Affymetrix) and each dataset was analyzed independently to reduce the confounding factors in the data analysis, such as batch effects and variabilities originated from array platforms. It was reported that the genetic basis of carcinogenesis involves a process of acquiring multiple genetic mutations in epithelial cells resulting in an activated stroma^26^. Considering this fact as well as the complexity of tumor tissues comprised of epithelial, stromal and immune cells, we analyzed data from laser micro-dissected epithelial samples from carcinoma cell population (CEPI) and healthy ovarian surface epithelial (OSE) tissues, only. Consequently, analysis of gene expression datasets from six independent studies associated with ovarian serous carcinoma by comparing gene expression levels between CEPI and OSE samples resulted with 698 mutual DETs, which were overrepresented with cell cycle, cancer signaling, drug and amino acid metabolisms.

The co-expression networks constructed around mutual DEGs in CEPI samples (CNC) and in OSE samples (CNO) represented observable differences in topological properties such as network size, density and clustering coefficient, and dispersed around different hub genes. Though the number of genes exhibiting co-expression pattern was almost two-fold higher in healthy state (i.e., CNO), network connectivity was higher in CNC. Consequently, a distinct tendency in gene expression correlations within diseased and healthy states was observed: the communication among genes decreases in diseased state; however, higher connectivity was developed between the genes.

Topological analysis of the CNC and CNO networks resulted with several modules. Among those, we identified a group of genes (84 genes) among mutual DEGs with strongly correlated co-expression pattern in CEPI samples, but not in OSE samples. The cooperation among these genes might be activated in response to the alteration of state, i.e., tumorigenesis. These genes may have a cooperative role in tumorigenesis process or this cooperative behavior may be a response of cells to the tumorigenesis process. In addition, the module could separate samples (patients) with long and short survival in ovarian cancer, and the death rate between groups was approximately twice, and at least 1.2 times. Therefore, this gene module represents a significant potential of “systems biomarkers” for therapeutics and prognosis in ovarian cancer.

Interestingly, the majority of these differential co-expressed genes was uncharacterized or encoding proteins taking roles in distinct signaling and metabolic pathways. Signaling proteins were mostly associated with signaling by GPCR. Dysregulation of G-protein and GPCR signaling leads to the initiation and progression of malignant tumor and their metastatic spread. More specifically mitogen-activated protein kinases (MAPK) signaling pathways such as Interleukin receptor SHC, PI3K-Akt, and Ras-Raf-Mek-Erk signaling, in which defect or alteration cause uncontrolled growth in terms of tumorigenesis^27–29^. Thus, it was speculated that module genes of these pathways might be considered as tumorigenesis related genes in ovarian cancer. The metabolism associated genes were encoding enzymes of energy, amino acid and protein metabolisms as expected, and represent potential for metabolic markers in ovarian cancer therapy.

The dense co-expression pattern of this gene set (CMC) may be as a result of the localization of the genes on the same chromosome or their transcription may be controlled through common transcriptional regulatory elements. When the chromosomal locations of the genes were analyzed, we observed that the module genes were distributed to almost all human chromosomes at different ratios. On the other hand, the transcription of the majority (89%) of module genes were regulated by six TFs, especially GATA2, YBX1 and AR. Unlike TFs, miRNAs represented a diverse regulatory pattern, where module genes were regulated by distinct miRNAs. This observation supports the hypothesis that TFs play general roles in transcriptional regulatory mechanisms; however, miRNAs take more specific duties^7^.

Screening the module genes across nine tumor tissues (ovarian, breast, cervical, prostate, lung, colorectal, pancreatic, thyroid, and leukemia) indicated distinct expression patterns of the CMC in different tumor tissues and strongly suggested that CMC is specific to ovarian cancer and the module genes play an important role in epithelial cell originated tumorigenesis.

The grand challenge in ovarian cancer research is that the origin of epithelial ovarian cancer is not clearly defined. Recent studies suggested that ovarian cancer may originate from precursor lesions located in the fallopian tubal epithelium^30^. This issue should be also considered in further researches. However, harboring a stem cell niche, OSE differs from the other differentiated epithelial cells, and malignant transformation may be explained by the presence of stem cell niches in those areas^31^. From this point of view, the results of the present study may have important implications for understanding ovarian cancer pathogenesis. Future research investments in experimental validations of corresponding disease biomarkers might provide new insights on ovarian cancer prognosis and treatment strategies that continue to place a significant burden on global health.

## Conclusions

Our study has demonstrated the presence of a novel prognostic gene module, which was differentially co-expressed and predictive of patient survival in ovarian cancer. Moreover, genes of the co-expressed module were co-regulated by a certain number of TFs and various miRNAs in an integrated manner, but did not represent a common regulatory pattern across different cancer types, and therefore the co-expressed and co-regulated module was specific to ovarian cancer. The proposed gene module will enable valuable insights through understanding the tumor formation and progression in ovarian epithelial cells. The set of genes in the module could be considered as systems biomarkers which may be used for screening or therapeutic purposes in ovarian carcinoma; more efforts are required to experimental and clinical validation of the findings.

## Methods

### Gene expression datasets

Taking into account the heterogeneous nature of ovarian cancer, we focused on micro-dissected epithelial samples from high grade serous ovarian carcinoma^32–37^. As a result of an extensive screening on ovarian cancer associated transcriptome datasets, six datasets (GSE14407^32^, GSE18521^33^, GSE23391^34^, GSE27651^35^, GSE38666^36^, and GSE40595^37^) that included laser micro-dissected epithelial samples from carcinoma cell population (CEPI) and healthy ovarian surface epithelial (OSE) tissues were considered. The raw data consisting of 191 samples (140 CEPI and 51 OSE samples) were obtained from the NCBI Gene Expression Omnibus (GEO)^38^, which is a public functional genomics data repository supporting MIAME-compliant data submissions.

### Identification of differentially expressed genes

To characterize differentially expressed genes (DEGs), each dataset was normalized by means of the Robust Multi-Array Average (RMA) expression measure^39^ as implemented in the “affy” package^40^ of R/Bioconductor platform (version Rx64 3.3). DEGs were identified from the normalized log-expression values using the multiple testing option of LIMMA (linear models for microarray data)^41^. Benjamini-Hochberg’s method was used to control the false discovery rate. An adjusted p-value threshold of 0.01 with a fold-change cutoff of 2 was used to determine the statistical significance of differential expression.

### Construction of co-expression networks in diseased and healthy states

Mutual DEGs of all datasets were determined. Expression profiles of CEPI and OSE samples were separated, and two new data subsets (consisting of 140 and 51 samples, respectively) were constructed using the expression profiles of mutual DEGs within diseased (CEPI) and healthy (OSE) states. To eliminate batch effects and ensure a similar empirical distribution of each array, both data subsets were normalized via RMA expression measure. In case of the repetitive DEGs, the mean expression values were computed and employed in further analyses. We computed the Pearson correlation coefficient (PCC) of the expression profiles between every pair of DEGs in each dataset. The resultant PCCs were approximately normally distributed in each case. A PCC cut-off, corresponding to p-value < 0.05, was determined and employed to identify statistical significance of the pairwise correlations. We established two co-expression networks, CNC and CNO, representing diseased and healthy states (i.e., CNC: co-expression network in CEPI samples, CNO: co-expression network in OSE samples), between the significantly co-expressed DEGs.

### Determination of network modules and their differential co-expression

To identify network modules of these networks, co-expression networks were analyzed using MCODE plugin of Cytoscape (v.2.8.3)^42^. Ranking of modules was based on MCODE scores (i.e., average connectivity). In selection of the modules for further analyses, modules with at least 10 nodes (genes), average connectivity ≥10 and network density ≥0.80 were considered. Since they might give important clues on disease pathogenesis and prognosis, we focused on modules of CNC, and co-expression pattern of these modules were also investigated in healthy state (i.e., within the CNO). Modules with significantly altered topological metrics (i.e., network density and clustering coefficient) were considered as differentially co-expressed modules between diseased and healthy states.

### Topological and functional enrichment analyses

Local and global topological features of networks and their modules were represented by several metrics, including degree, betweenness connectivity, network density, and clustering coefficient, and were determined via NetworkAnalyzer^43^ and Cytohubba^44^ plugins of Cytoscape (v.2.8.3). The dual-metric approach^12, 14^ incorporating degree as a local metric and betweenness centrality as a global metric was employed at identification of hub molecules, and top five molecules in terms of any of the metrics were presented as hubs.

Pathway enrichment analyses of gene sets (within networks or modules) were performed through DAVID bioinformatics tool (v.6.8)^45^ using KEGG^46^, and Reactome^47^ as the data sources. P-values were determined through Fisher Exact test and adjusted via Benjamini-Hochberg’s method. A threshold of adjusted p-value < 0.05 was used to determine the statistical significance of enrichment results. In addition, to characterize the pathway or process annotation of each gene, we manually searched GeneCards Human Gene Database^48^.

### Prognostic power analysis

We extracted gene expression profiles of module genes from the ovarian cancer dataset (GSE18521^33^), which supports clinical information for survival analysis. Then, PCA was performed based on gene expression profiles of 84 module genes, and samples were separated into two clusters using k-means algorithm taking into consideration the first three principle components. The survival time statistics were calculated by log-rank test and visualized in Kaplan-Meier survival curve. Cox (proportional hazards) regression was also employed to estimate hazard ratio.

### Identification of transcriptional regulatory networks

To investigate the common transcriptional regulators (i.e., TFs and/or miRNAs) of genes within the differentially co-expressed module (CMC), experimentally validated transcriptional regulatory interactome data^7^ were updated via addition of TF-target gene interactions from the latest version of HTRI database^49^ and miRNA-target gene interactions from mirTarBase (ver.6.0)^50^. For each component of CMC, the number of TFs and miRNAs regulating the mentioned gene was counted, and the mutual TFs and miRNAs regulating the significant portions of the module components were determined.

### Screening the differential expression of the module in different tumor types

Genes of the differentially co-expressed module in CEPI samples were investigated across nine tumor tissues (i) to determine specificity of the module to ovarian cancer, and (ii) to analyze the expression pattern of the module genes in different tumor tissues. For these purposes, gene expression datasets associated with ovarian cancer (GSE19352^51^, GSE26712^52^), breast cancer (GSE15852)^53^, cervical cancer (GSE9750)^54^, prostate cancer (GSE6919)^55^, lung cancer (GSE19804)^56^, colorectal cancer (GSE32323)^57^, pancreatic cancer (GSE28735)^58^, thyroid cancer (GSE3467)^59^ and leukemia (GSE26725)^60^ were employed, and statistically significant DETs were identified (as described before) comparing tumor vs. healthy tissues. Module genes were screened within the identified DEGs for each cancer type, and the expression pattern of the module genes in different tumor tissues (comprising of stroma, immune and epithelial cells) were analyzed.

## Electronic supplementary material

Supplementary Information
