# Supplementary material for: Differential co-expression analysis reveals a novel prognostic gene module in ovarian cancer
Source: Sci Rep. 2017 Jul 10;7:4996. doi: 10.1038/s41598-017-05298-w (PMC5504034; doi:10.1038/s41598-017-05298-w)
Supplement: Supplementary file 1 — Supplementary Information [file 41598_2017_5298_MOESM1_ESM.pdf]

Differential co-expression analysis reveals a novel prognostic gene module in ovarian cancer

Esra Gov[1], Kazim Yalcin Arga[1]*

[1]Department of Bioengineering, Marmara University, 34722 Goztepe, Istanbul, Turkey

Esra Gov: esra.korurer@marmara.edu.tr

Kazim Yalcin Arga: kazim.arga@marmara.edu.tr

Supplementary Table S1: Modules of the co-expression networks (CNC and CNO) and their topological parameters.

Module name	# nodes	# edges	Density	Average connectivity
CMC Module 1	84	3078	0.88	38.50
CMC Module 2	14	83	0.91	5.93
CMC Module 3	5	9	0.90	1.80
CMC Module 4	10	15	0.33	1.50
CMC Module 5	4	5	0.83	1.25
CMC Module 6	4	5	0.83	1.25
CMC Module 7	3	3	1	1
CMC Module 8	3	3	1	1
CMC Module 9	3	3	1	1
CMC Module 10	3	3	1	1
CMO Module 1	52	1178	0.89	22.65
CMO Module 2	37	519	0.78	14.03
CMO Module 3	14	83	0.91	5.93
CMO Module 4	26	90	0.28	3.42
CMO Module 5	14	46	0.51	3.29
CMO Module 6	6	15	1	3
CMO Module 7	14	29	0.32	2.08
CMO Module 8	10	20	0.44	2.00
CMO Module 9	7	13	0.62	1.85
CMO Module 10	5	9	0.90	1.80
CMO Module 11	5	8	0.80	1.60
CMO Module 12	4	5	0.83	1.25
CMO Module 13	4	5	0.83	1.25
CMO Module 14	3	3	1	1
CMO Module 15	3	3	1	1
CMO Module 16	3	3	1	1
CMO Module 17	3	3	1	1
CMO Module 18	3	3	1	1
CMO Module 19	3	3	1	1
CMO Module 20	3	3	1	1
CMO Module 21	3	3	1	1
CMO Module 22	3	3	1	1

Supplementary Table S2: Differentially co-expressed genes in ovarian cancer and their connectivity in CMC and CMO.

Gene Symbol	Gene name	Connection in CMC	Connection in CMO
AAA1	Aortic aneurysm, familial abdominal 1	83	2
BTRC	Beta-transducin repeat containing E3 ubiquitin protein ligase	83	9
C9orf93	Chromosome 9 Open Reading Frame 93	83	1
CTXN3	Cortexin 3	83	2
CYP19A1	Cytochrome P450, family 19, subfamily A, polypeptide 1	83	11
DIRAS2	DIRAS family, GTP-binding RAS-like 2	83	8
GABRG1	Gamma-aminobutyric acid (GABA) A receptor, gamma 1	83	3
GADL1	Glutamate decarboxylase-like 1	83	0
MTUS2	Microtubule associated tumor suppressor candidate 2	83	8
NEGR1	Neuronal growth regulator 1	83	7
PCDP1	Primary ciliary dyskinesia protein 1	83	3
PDE5A	Phosphodiesterase 5A, cGMP-specific	83	1
PDE8B	Phosphodiesterase 8B	83	2
PKD1L2	Polycystic kidney disease 1-like 2	83	1
PPM1E	Protein phosphatase, Mg2+/Mn2+ dependent, 1E	83	1
PRG4	Proteoglycan 4	83	0
SLC3A1	Solute carrier family 3 (amino acid transporter heavy chain), member 1	83	5
SNCAIP	Synuclein, alpha interacting protein	83	2
SV2C	Synaptic vesicle glycoprotein 2C	83	6
ZIC4	Zic family member 4	83	1
ZNF566	Zinc finger protein 566	83	13
CELF4	CUGBP, Elav-like family member 4	82	0
CNR1	Cannabinoid receptor 1 (brain)	82	0
FBXL3	F-box and leucine-rich repeat protein 3	82	3
LHX9	LIM homeobox 9	82	0
PAPOLG	Poly(A) polymerase gamma	82	0
SVEP1	Sushi, von Willebrand factor type A, EGF and pentraxin domain containing 1	82	0
DSCAML1	Down syndrome cell adhesion molecule like 1	81	0
IQGAP3	IQ motif containing GTPase activating protein 3	81	2
CDC14A	Cell division cycle 14A	80	8
CNIH3	Cornichon family AMPA receptor auxiliary protein 3	80	4
CNRIP1	Cannabinoid receptor interacting protein 1	80	0
SH3RF2	SH3 domain containing ring finger 2	80	6
BUB1	BUB1 Mitotic Checkpoint Serine/Threonine Kinase	79	6
DTNA	Dystrobrevin, alpha	79	0
MED23	Mediator complex subunit 23	79	0
PIP5K1B	Phosphatidylinositol-4-phosphate 5-kinase, type I, beta	79	6
PRSS35	Protease, serine, 35	79	0
C20orf112	Chromosome 20 open reading frame 112	78	6
PLCE1	Phospholipase C, epsilon 1	78	0

EPB42	Erythrocyte membrane protein band 4.2	77	0
PTGDR	Prostaglandin D2 receptor (DP)	77	7
KCNB1	Potassium voltage-gated channel, Shab-related subfamily, member 1	74	4
SOBP	Sine oculis binding protein homolog (Drosophila)	74	3
ZNF471	Zinc finger protein 471	74	2
CDC25C	Cell division cycle 25C	73	1
HSD17B2	Hydroxysteroid (17-beta) dehydrogenase 2	73	0
NEDD4L	Neural precursor cell expressed, developmentally down-regulated 4-like, E3 ubiquitin protein ligase	73	0
SOX6	SRY (sex determining region Y)-box 6	73	3
GATM	Glycine amidinotransferase (L-arginine:glycine amidinotransferase)	72	0
OLFML1	Olfactomedin-like 1	72	0
DENND5B	DENN/MADD domain containing 5B	71	3
C4orf32	Chromosome 4 open reading frame 32	70	0
FAM153B	Family with sequence similarity 153, member B	70	5
DYNC2H1	Dynein, cytoplasmic 2, heavy chain 1	69	5
ELAVL2	ELAV Like Neuron-Specific RNA Binding Protein 2	69	0
PEX5L	Peroxisomal biogenesis factor 5-like	69	0
GNG4	Guanine nucleotide binding protein (G protein), gamma 4	68	0
KRT73	Keratin 73	68	0
SEL1L2	Sel-1 suppressor of lin-12-like 2 (C. elegans)	68	2
DIXDC1	DIX domain containing 1	67	10
MAOB	Monoamine oxidase B	67	0
IGHD	Immunoglobulin heavy constant delta	65	0
LEPR	Leptin receptor	65	0
RAB31	RAB31, member RAS oncogene family	65	0
GNG11	Guanine nucleotide binding protein (G protein), gamma 11	64	0
KLHDC1	Kelch domain containing 1	64	0
DTWD1	DTW domain containing 1	63	2
NR0B1	Nuclear receptor subfamily 0, group B, member 1	63	1
AMDHD1	Amidohydrolase domain containing 1	62	2
CLEC4M	C-type lectin domain family 4, member M	61	0
PMP22	Peripheral myelin protein 22	61	3
ADCYAP1	Adenylate cyclase activating polypeptide 1	60	1
HM13	Histocompatibility (minor) 13	60	0
CMYA5	Cardiomyopathy associated 5	59	0
MCTP2	Multiple C2 domains, transmembrane 2	59	0
SCAF4	SR-Related CTD-Associated Factor 4	58	7
MMP28	Matrix metallopeptidase 28	57	2
N4BP2L1	NEDD4 binding protein 2-like 1	57	0
PIK3C3	Phosphatidylinositol 3-kinase, catalytic subunit type 3	57	4
PTGER3	Prostaglandin E receptor 3 (subtype EP3)	57	0
TRAF4	TNF receptor-associated factor 4	57	3
CASC5	Cancer susceptibility candidate 5	56	3
SYT4	Synaptotagmin IV	50	0
